# Interaction of silver nanoparticles with Tacaribe virus

**DOI:** 10.1186/1477-3155-8-19

**Published:** 2010-08-18

**Authors:** Janice L Speshock, Richard C Murdock, Laura K Braydich-Stolle, Amanda M Schrand, Saber M Hussain

**Affiliations:** 1 Applied Biotechnology Branch, Human Effectiveness Directorate, 711th Human Performance Wing, U.S. Air Force Research Laboratory, 2729 R Street, Wright-Patterson Air Force Base, OH, 45433-5707, USA

## Abstract

**Background:**

Silver nanoparticles possess many unique properties that make them attractive for use in biological applications. Recently they received attention when it was shown that 10 nm silver nanoparticles were bactericidal, which is promising in light of the growing number of antibiotic resistant bacteria. An area that has been largely unexplored is the interaction of nanomaterials with viruses and the possible use of silver nanoparticles as an antiviral agent.

**Results:**

This research focuses on evaluating the interaction of silver nanoparticles with a New World arenavirus, Tacaribe virus, to determine if they influence viral replication. Surprisingly exposing the virus to silver nanoparticles prior to infection actually facilitated virus uptake into the host cells, but the silver-treated virus had a significant reduction in viral RNA production and progeny virus release, which indicates that silver nanoparticles are capable of inhibiting arenavirus infection *in vitro*. The inhibition of viral replication must occur during early replication since although pre-infection treatment with silver nanoparticles is very effective, the post-infection addition of silver nanoparticles is only effective if administered within the first 2-4 hours of virus replication.

**Conclusions:**

Silver nanoparticles are capable of inhibiting a prototype arenavirus at non-toxic concentrations and effectively inhibit arenavirus replication when administered prior to viral infection or early after initial virus exposure. This suggests that the mode of action of viral neutralization by silver nanoparticles occurs during the early phases of viral replication.

## Background

The family *Arenaviridae *is composed of 18 different species of viruses divided into two antigenic groups, the Old World and New World (Tacaribe complex) groups. The Tacaribe complex, in addition to Tacaribe virus (TCRV), includes the viral hemorrhagic fever-inducing viruses Junin, Machupo, Guanarito, and Sabia. Close antigenic relationships are observed between TCRV, a non-human pathogen, and the category A arenaviruses [1]. TCRV is a biochemically and serologically close relative of Junin and Guanarito viruses but has a low pathogenic potential for humans and is more easily amenable to laboratory study [2]. Arenaviruses are highly fatal and currently have no available vaccines and there is little research to support efficacy of antivirals [3].

Current technology offers the possibility of generating new types of nanostructured materials (often 100 nm or smaller) with designed surface and structural properties that render them reactive and able to bind proteins avidly [4-6]. Silver nanoparticles (Ag-NPs) have received considerable attention for biological applications and recently it was shown that highly concentrated and nonhazardous nanosized silver particles can be easily prepared in a cost-effective manner and possess antimicrobial properties [7]. The interaction of NPs with microorganisms is an expanding field of research; however, little effort has been done to determine the interaction of metal NPs with viruses, although recent studies have shown that replication of HIV-1 [8] and Monkeypox virus [9] can be inhibited by Ag-NPs.

This study focuses on understanding the interaction of Ag-NPs with arenaviruses and the effects on viral replication. Arenaviruses are enveloped viruses with an ambisense RNA genome, which is structurally very different than HIV or poxviruses [3]. The arenaviruses also replicate differently than HIV or poxviruses, but have a similar replication cycle to other important viruses, such as filoviruses or orthomyxoviruses, both of which are also enveloped, RNA viruses. Therefore it is important to determine how Ag-NPs interact with enveloped, RNA viruses to conclude if they are virucidal against other virus families as well as HIV and poxviruses. Two types of Ag-NPs are used in the subsequent study, uncoated (Ag-NP) and polysaccharide-coated (PS-Ag), to assess the impact of biocompatible coatings on viral replication since the addition of a polysaccharide coat onto Ag-NPs has been shown to decrease their toxicity in mammalian cells [10]. Our findings demonstrate that the interaction of TCRV with Ag-NPs prior to cellular exposure results in a decrease in viral infectivity with 10 and 25 nm Ag-NPs and the addition of a polysaccharide coating on the Ag-NPs renders them slightly less effective at inhibiting viral replication.

## Results

### Biocompatibility of Ag-NPs in Vero cells

**Figure 1:**
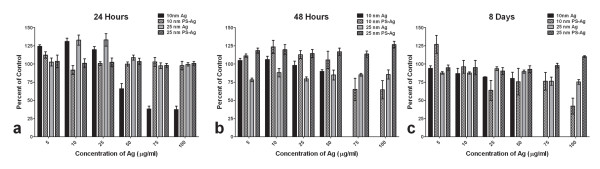
**Biocompatibility of Ag-NPs in Vero cells**. Vero cells were exposed to Ag-NPs for 24 hours (A), 48 hours (B), or 8 days (C) and the cell viability was determined using a standard MTS assay. The effects of the Ag-NPs on cellular viability are expressed as percent of control (untreated Vero cells) with error bars representing standard error of the mean (S.E.M.).

After a 24 hour exposure, a 25% decline in cell viability was observed in Vero cells exposed to 50 μg/ml of 10 nm uncoated Ag-NPs (Fig 1a). Treatments with 10 nm uncoated Ag-NPs at 75 and 100 μg/ml resulted in a 60% reduction in cell viability (Fig 1a). There was no further reduction in cell viability in the 50 μg/ml dose, but the cells treated with 75-100 μg/ml died off by day 2 (Fig 1b, c). The 10 nm PS-Ag had no significant effects on the Vero cells in the first 24 hours (Fig 1a) but the 75 and 100 μg/ml doses demonstrated a 25% reduction in viability after 48 hours (Fig 1b, c) suggesting an instability of the coating. Doses lower than 50 μg/ml of either 10 nm NP had little effect on Vero cell viability (Fig 1). There was little cytotoxicity observed in Vero cells treated with either the uncoated or polysaccharide-coated 25 nm Ag-NPs (Fig 1).

### Ag-NP interactions with TCRV

Prior to determining the effect of Ag-NPs on TCRV infection, we wanted to conclude whether or not there are direct interactions between the Ag-NPs and the virus. Table 1 represents a comparison of the Ag-NP size distributions of the particles dried onto grids and imaged using transmission electron microscopy (TEM; Table 1a) and the size in solution determined by dynamic light scattering (DLS; Table 1b-d). When added to the cell culture media DMEM at 50 μg/ml, the Ag-NPs aggregated to almost a micron in size with the 10 nm uncoated particles showing the greatest amount of aggregation (Table 1b). Interestingly when the Ag-NPs were incubated with TCRV at NP doses of 10 or 50 μg/ml, a mean size of 300-500 or 400-900 nm, respectively, was observed (Table 1c, d), which is smaller than the aggregates formed by the Ag-NPs alone. As with the aggregation of Ag-NPs in solution, the largest aggregates formed with the virus are with the 10 nm uncoated Ag-NPs. The Ag-NP-TCRV aggregate size is between the Ag-NP aggregates without virus and the size of the virus (165 nm) in solution. This suggests that the Ag-NPs are interacting with TCRV and that the binding affinity for the viral proteins may be stronger than that of the NPs with each other.

**Table 1:** Nanoparticle size distributions dry and in solution.

	10 nm	10 nm PS	25 nm	25 nm PS
**(a) NP TEM size (SD)**	10.20 (1.70)	9.48 (4.29)	27.47 (9.06)	25.98 (8.38)
**(b) NPs in DMEM (PDI)**	1440 (0.277)	658 (0.261)	830 (0.214)	986 (0.217)
**(c) 10 μg NP + TCRV (PDI)**	503 (0.334)	481 (0.444)	326 (0.366)	305 (0.344)
**(d) 50 μg NP + TCRV (PDI)**	974 (0.465)	756 (0.462)	524 (0.388)	426 (0.403)

The size distributions of the four Ag-NPs were determined using TEM size measurements with mean particle size and standard deviation (A), and DLS measurements of Ag-NPs alone in DMEM (B), 10 μg of Ag-NPs incubated with TCRV for 1 hour (C), or 50 μg of Ag-NPs incubated with TCRV for 1 hour (D) with average size and polydispersity index.

### TCRV neutralization by Ag-NPs

**Figure 2:**
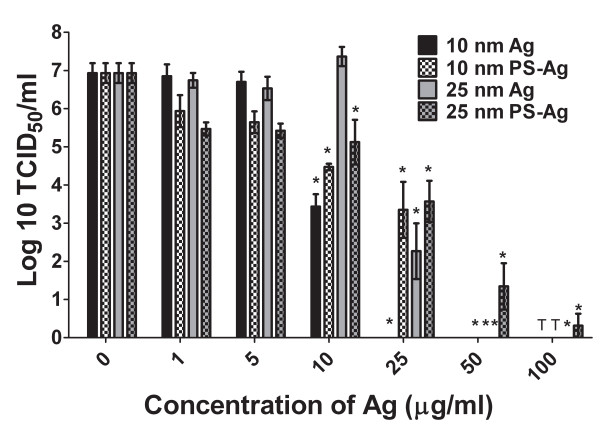
**TCRV replication following exposure to Ag-NPs**. TCRV was treated with uncoated and PS-coated 10 and 25 nm Ag-NPs for 1 hour. Treated or control virus suspensions were used to infect Vero cells for 8 days. Progeny virus was recovered from the cell culture supernatant and was quantified using a standard tissue culture infectious dose assay. Progeny virus titers are expressed as the mean log_10 _TCID_50_/mL +/- S.E.M. (T = concentration of nanoparticles was toxic at this dose; *p < 0.05; student's t test; n = 6).

When incubated with the virus 1 hour prior to infection of the Vero cells, the 10 nm uncoated Ag-NP resulted in the most dramatic reduction in TCRV virus titer with approximately 50% reduction in progeny virus titer at 10 μg/ml and no detectable progeny virus at 25 μg/ml or greater (Fig 2). The PS-Ag (both 10 and 25 nm) also had a significant reduction in virus titer at 10 and 25 μg/ml, and was almost undetectable at concentrations of 50 μg/ml and greater (Fig 2). However, the 25 nm PS-Ag had 3 out of 6 replicates with detectable virus titers at 50 μg/ml and 1 out of 6 trials at 100 μg/ml. The 25 nm uncoated Ag-NP did not significantly reduce TCRV replication at concentrations lower than 25 μg/ml, but there was no detectable virus titer at 50 or 100 μg/ml with these particles (Fig 2).

### Ag-NP treated TCRV interactions with the Vero cells

**Figure 3:**
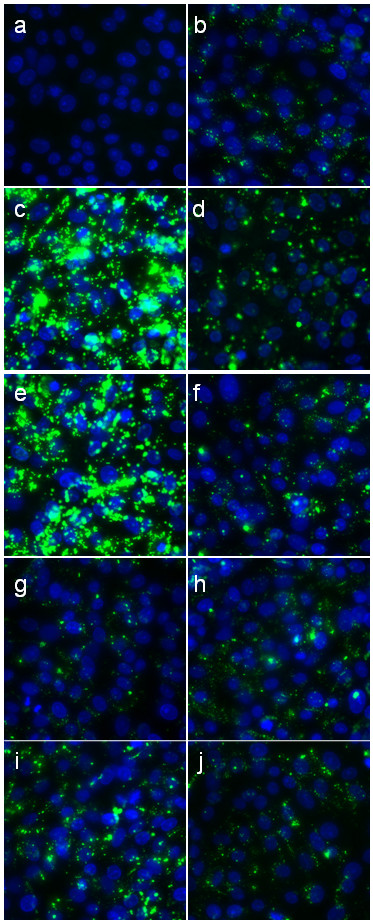
**Confocal imaging of untreated and Ag-NP-treated TCRV in Vero cells**. TCRV was labeled with a fluorophore that excites at a wavelength of 488 nm and was treated with Ag-NPs for 1 hour. Treated or control virus was used to infect Vero cells for 4 hours. The supernatant was removed and the cells were washed 2 times with PBS to remove non-adherent virus and were fixed in 3% paraformaldehyde. The nuclei were stained with Hoechst (blue) and the images were taken using spinning disc Confocal microscopy with the pictures representative of collapsed z-stacks of sections through the cell (15 sections). (a) Vero cells alone (b) TCRV in Vero cells (c) TCRV + 10 nm Ag 50 μg/ml (d) TCRV + 10 nm Ag 10 μg/ml (e) TCRV + 10 nm PS-Ag 50 μg/ml (f) TCRV + 10 nm PS-Ag 10 μg/ml (g) TCRV + 25 nm Ag 50 μg/ml (h) TCRV + 25 nm Ag 10 μg/ml (i) TCRV + 25 nm PS-Ag 50 μg/ml (j) TCRV + 25 nm PS-Ag 10 μg/ml (representative picture of 3 separate trials).

Fluorescently-tagged TCRV (green) was incubated with Ag-NPs for 1 hour and then was used to infect Vero cells for 4 hours. This time-point was chosen since it was ample time for cellular interaction and internalization of the virus, but the fluorescent tag diminished in intensity for longer incubations. Following the incubation, confocal microscopy using serial sections through the cell was used to image the cells with the figure representing a collapsed z-stack of these sections. Vero cells infected with untreated TCRV showed a good amount of virus internalized and was well dispersed throughout the monolayer (Fig 3b). TCRV treated with either uncoated or PS-coated 10 nm Ag-NPs at 50 μg/ml interacted with and internalized much more efficiently into the cell than the untreated control (Fig 3c, e versus 3b). The 10 μg/ml 10 nm treated viruses also showed a slight increase in uptake into the cells but not as dramatic and the 50 μg/ml dose (Fig 3d, f). The 25 nm PS-Ag 50 μg/ml treated TCRV had an increase in internalization comparable to the 10 nm 10 μg/ml treatments (Fig 3i). The 10 μg/ml dose of the 25 nm PS-Ag and either dose of the 25 nm uncoated Ag-NPs had minimal effect on the cellular uptake of the virus (Fig 3g, h, j). Hoechst was used to label the nuclei of the cells.

### Ag-NP treated TCRV internalization into Vero cells

**Figure 4:**
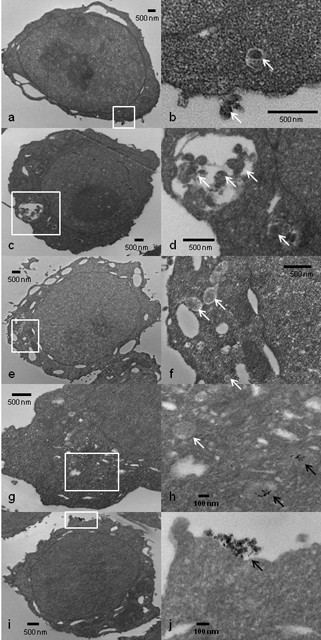
**TEM of TCRV internalization into Vero cells**. TEM micrographs depict Vero cells infected with untreated TCRV (a, b), 10 nm (uncoated, 50 μg/ml) treated TCRV (c, d), or 25 nm (uncoated, 50 μg/ml) treated TCRV (e, f) with b, d, and f being zoomed-in images of the white squares of a, b, and c. Images g and h depict virus and the Ag-NPs localizing within the same cell, and i and j depict the interaction of TCRV with the Ag-NPs outside of the cell. White arrows are pointing towards the virus and black arrows show the Ag-NPs.

While the z-sections through the confocal images suggested internalization of TCRV into Vero cells even when treated with Ag-NPs, we wanted to confirm that the virus is indeed entering the cell using TEM. Fig 4a and 4b depicts untreated TCRV inside of Vero cells. The 10 nm Ag-NP (uncoated, 50 μg/ml) treated TCRV is not only able to enter the cell but it also appears to have several virions enter into the same endosome (Fig 4c, d), which explains the large fluorescent aggregates observed in the confocal images (Fig 3c, e). TCRV treated with 25 nm Ag-NPs (uncoated, 50 μg/ml) again is effectively internalized into Vero cells (Fig 4e, f) but they appear to enter individual endosomes (Fig 4f). TEM micrographs were also added that showed Ag-NPs internalized into the same cell as a TCRV virion (Fig 4g, h) and the Ag-NPs interacting with the virus outside of the cell (Fig 4i, j).

### Viral RNA replication

**Figure 5:**
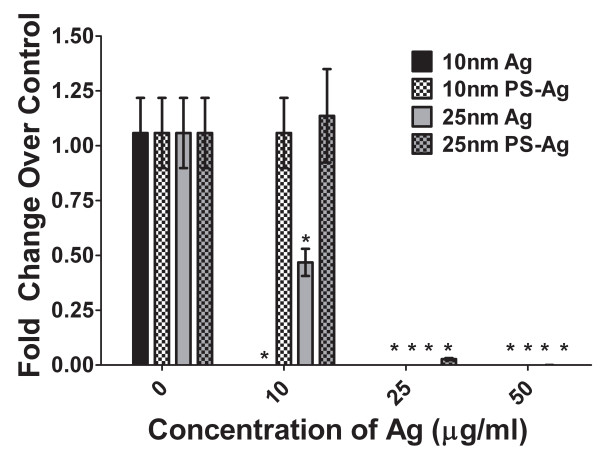
**S segment quantitative real time PCR analysis**. TCRV was treated with uncoated and PS-coated 10 and 25 nm Ag-NPs for 1 hour. Treated or control virus was used to infect Vero cells for 4 days. The supernatant was removed and the cells were washed 2 times with PBS to remove non-adherent virus. RNA was isolated from the Vero cells and S segment gene expression was determined using qRT-PCR with Sybr green. Changes in S segment gene expression are expressed as fold change over untreated TCRV infection with error bars representing the S.E.M. and statistical analysis comparing the untreated control to each of the treatment groups. (*p < 0.05; student's t test; n = 6).

Since we determined that the virus is entering the cell but progeny virus production is impaired following pre-treatment with Ag-NPs, we wanted to determine whether the Ag-NPs inhibited viral RNA replication. Therefore we examined the amount of S segment gene expression in TCRV-infected Vero cells with and without Ag-NP treatment. Arenaviruses have 2 strands of RNA, the S and L segments. The S segment is the segment that encodes the nucleoprotein and GP1 and 2 proteins, which are early proteins and therefore are detectable early in viral replication. At 25 and 50 μg/ml, all 4 Ag-NPs tested had a dramatic reduction in the amount of S segment expression as compared to the untreated TCRV control (Fig 5). This correlated with the significant reduction in progeny virus produced following the Ag-NP treatments. There was also a significant decrease in the S segment gene expression of the Vero cells infected with TCRV treated with 10 μg/ml of 10 and 25 nm uncoated Ag-NPs (Fig 5).

### Post-infection Ag-NP treatment

Given that the replication of viral genes is inhibited in Ag-NP-treated virus infections, we propose that the Ag-NPs must be acting on early stages of virus replication to prevent infection.

**Figure 6:**
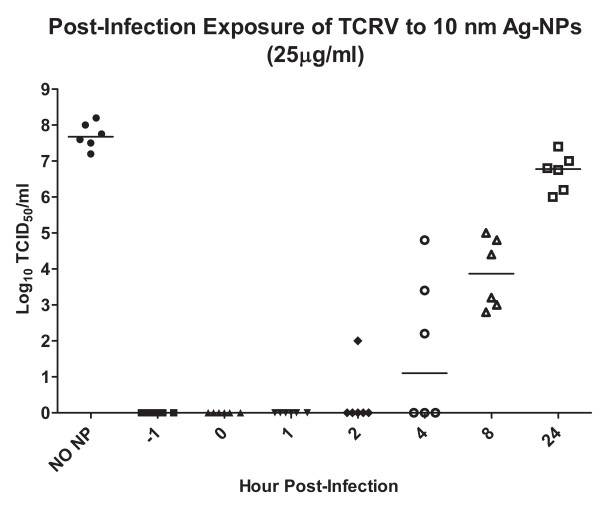
**Vero cells were infected with TCRV and were exposed to 10 nm Ag-NPs at 25 μg/ml at 1, 2, 4, 8, and 24 hours post-infection or the Ag-NPs were added concurrently with the TCRV (0 hour)**. The -1 hour reflected the pre-exposure scenario of figure 1 and was used as a control. The progeny virus produced was harvested 8 days post-infection and is expressed as the mean log_10 _TCID_50_/mL. All treatments were significantly lower than the "No NP" control as determined by the student's t test with n = 6.

To conclude that the Ag-NPs are inhibiting early steps of viral replication and to determine how effective the Ag-NPs would be as a therapeutic agent, we administered the uncoated 10 nm Ag-NPs at a dose of 25 μg/ml at various time-points post-infection. This dose with this NP was benign to the Vero cells, as determined by a mitochondrial function assay (Fig 1), and was completely effective at inhibiting viral replication when administered prior to infection (Fig 2). A pre-treatment at -1 hour was used as a control for Ag-NP inhibition and then the Ag-NPs were added to virally-infected Vero cells at 0, 1, 2, 4, 8, and 24 hours post-infection. At the -1, 0, and 1 hour time-points, there was no detectable progeny virus produced (Fig 6). There was only one sample out of six trials that had any detectable virus production when the Ag-NPs were administered 2 hours post-infection (Fig 6). However, at 4 hours post-infection, only 50% of the trials showed complete inhibition of viral replication, and the 12 and 24 hours time-points had significantly lower end virus titers than the untreated control, but still had an elevated viral titer after the 8-day replication cycle (Fig 6). Alternatively, Vero cells were also pre-treated with Ag-NPs for 1, 24, or 48 hours prior to infection with TCRV. However, there was no detectable decline in virus titers or vRNA replication when the Ag-NPs were administered to the cells pre-infection (data not shown).

## Discussion

The goal for this study was to assess the interaction of Ag-NPs with a prototypical arenavirus, TCRV, and to determine if Ag-NPs could cause a significant decrease in progeny virus production at a concentration that is relatively benign to the host cell. Because of the capacity for person-to-person aerosol transmission, the lack of diagnostic testing, and limited therapeutic options, the arenaviruses are included in the category A list of potential bioweapons [11]. Although there is an experimental vaccine for Junin, and ribavirin has shown some efficacy, the vaccine is yet approved for human use and viruses rapidly acquire resistance to antivirals, which could leave people vulnerable to arenavirus diseases [3]. Since Ag-NPs have been suggested to be an alternative anti-bacterial therapeutic, we wanted to determine if they could also be used to prevent arenavirus infection.

Our findings show that the uncoated 10 nm Ag-NPs are toxic to Vero cells at doses greater than 50 μg/ml (Fig 1). However when TCRV was treated with 50 and 25 μg/ml of these particles, i.e. nontoxic doses, there was no detectable progeny virus produced (Fig 2). Even at 10 μg/ml the 10 nm Ag-NP showed a significant reduction in progeny virus titer, and the 10 nm PS-Ag particles showed a similar trend but were not as effective (Fig 2). Significant toxicity of 10 nm PS-Ag was only observed in the Vero cells at doses of 100 μg/ml, but only 50 μg/ml was required for complete inhibition of virus replication (Fig 2). Again the 10 and 25 μg/ml doses of the 10 nm PS-Ag caused a significant reduction in viral replication, but it was not as effective as the treatment with the uncoated 10 nm particles (Fig 2). Therefore the polysaccharide coating may indeed protect the cell from the toxic effects of the Ag-NPs, but it also appears to interfere with the Ag-NP interaction with TCRV. The 25 nm particles, both PS-coated and uncoated, displayed limited toxicity in the Vero cells and they too inhibited viral replication at 50 μg/ml and showed a significant reduction in progeny virus production at 25 μg/ml. The inhibition by the 25 nm particles suggests that arenavirus replication is inhibited by Ag-NPs using a different mechanism than either HIV-1 or Monkeypox virus, both of which are inhibited by 10 nm, but not 25 nm Ag-NPs [8,9].

Previous research demonstrates that Ag-NPs preferentially bind to the gp120 knobs of HIV virus [8]. Our DLS data suggests that a similar trend may be occurring with TCRV, and the Ag-NPs are likely binding to the membrane glycoproteins of the virus. Since there are many cysteines located throughout the TCRV glycoproteins [12] and Ag-NPs readily bind to the thiol groups [13], which are found in cysteine residues, there is likely a strong interaction between the TCRV and Ag-NPs. This interaction will likely either prevent internalization of the virus by inhibiting glycoprotein-receptor interactions, or will internalize into the cell together and effect viral replication within the cell. The later statement appears to be the case since the confocal images depict internalization of the virus and this is confirmed with TEM. Ag-NPs have been shown to enter the cell via macropinocytosis or clathrin-mediated endocytosis [14]. TCRV and other arenaviruses also enter the cell via clathrin-mediated endocytosis via interaction with a cellular receptor [15]. The similarity of intracellular internalization mechanisms further suggests the potential for intracellular interactions, illustrated by TEM images that depict TCRV and Ag-NPs taken up into the same cell, even if the glycoprotein-Ag-NP interaction is not retained inside of the cell. Since the vRNA appears to be inhibited in Ag-NP-treated TCRV infections, this could indicate that the Ag-NPs could be interfering with the TCRV RNA-dependent RNA polymerase (L protein). Ag and Ag-NPs have been shown to be capable of inhibiting enzyme activity [16,17] and perhaps the Ag-NPs are inhibiting the enzymatic activity of the L protein when TCRV and Ag-NPs enter into the same cells. However, the virus may not even able to get to the RNA replication step. It is possible that the Ag-NPs may be able to prevent the virus uncoating in the endosome. An alternative theory to explain the viral neutralization capabilities of Ag-NPs could be that they are inferring with the zinc (Zn)-finger motif of the virus. It has been previously demonstrated that disulfide-based compounds are capable of interacting with the cysteine residues of the Zn-binding domains of Junin and TCRV, which leads to cross-linking of the protein and loss of the Zn [18]. Two arenavirus proteins, the nucleoprotein and the small Z protein, have been shown to possess Zn-binding motifs [19,20]. Garcia et al. concluded that virions treated with disulfide-based compounds are capable of entering the cell with the efficiency as untreated virions, but they are not able to synthesize viral proteins since they are inactivated by the compounds prior to cell entry [18]. This is very similar to what we are observing with the Ag-NPs, which also readily bind to cysteine residues. Since pre-treatment of the cells with Ag-NPs had no effect on viral replication it is plausible to assume that the Ag-NPs could be inactivating the virus prior to entry into the cell.

## Conclusion

TCRV is a prototype New World clade B arenavirus that shares structural and genomic homology with the South American hemorrhagic fever viruses Junin, Machupo, Guanarito, and Sabia [18]. Since these highly virulent hemorrhagic viruses share the same proteins and mechanism of replication as TCRV, the Ag-NPs will likely inhibit their replication as well. Due to the known toxicity of Ag-NPs in many human cell lines [10], and the short time limit of efficacy following infection, the Ag-NPs would likely make a more effective decontamination tool as opposed to an *in vivo *therapeutic agent. However, if the Ag-NPs do indeed facilitate the uptake of arenaviruses into the cell and inactivate the virus prior to cell entry, further studies should be performed to determine if Ag-NPs can prove to be an effective vaccine adjuvant.

## Materials and methods

### Cell cultures and virus propagation

Vero cells (CCL-81; ATCC, Manassas, VA) were maintained in Dulbecco's modified Eagle's Medium (DMEM; Biowhitaker, Basel, Switzerland) supplemented with 10% heat-inactivated fetal bovine serum (FBS; Gibco, Carlsbad, CA) and 1% Penicillin-Streptomycin (P/S; Invitrogen, Carlsbad, CA). Stocks of Tacaribe virus (TCRV; VR-1556; ATCC, Manassas, VA) were prepared by infecting the Vero cells at a low multiplicity of infection (MOI = 0.01) for 1 hour at 37°C in 5% CO_2_. Following virus absorption, unbound virus was removed and washed with phosphate buffered saline (PBS), and DMEM supplemented with 2% FBS and 1% PS was added to the cells. The virus was allowed to replicate for 8 days at 37°C in 5% CO_2_, which is when complete cytopathic effect was observed. Virus was recovered from the cell supernatant and the cellular debris was removed from the viral suspension via centrifugation at 580 × g for 15 minutes (Allegra 25R Centrifuge; Beckman Coulter, Fullerton, CA). The stocks were preserved with 1% bovine serum albumin (BSA; Sigma, St. Louis, MO) and stored at -80°C.

### Nanoparticles

The uncoated 25 nm Ag-NPs were a kind gift from Dr. Karl Martin (Novacentrix, Austin, TX) and the uncoated Ag-NPs at 10 nm were synthesized by Dr. Steven Oldenburg (NanoComposix, San Diego, CA). Polysaccharide-coated Ag-NPs (PS-Ag) 10 and 25 nm were a generous gift from Dr. Dan Goia (Clarkson University, Center for Advanced Materials Processing, Potsdam, NY). These PS-Ag NPs were synthesized by the reduction of Ag ions in solution by a polysaccharide (acacia gum), which leads to a polysaccharide surface coating [10]. All Ag-NPs used in this study were spherical in morphology and were diluted in deionized water to 1 mg/ml and were then further diluted in cell culture media. Characterization data, such as TEM images and size distribution, elemental analysis, and agglomerate size in solution (DMEM using DLS) were supplied with the NPs and confirmed in our laboratory [10].

### Nanoparticle treatment

Ag-NPs were diluted in DMEM and sonicated with a probe sonicator for dispersion. TCRV was added to the Ag-NP/DMEM mixture at a 1:40 dilution (5 × 10^4 ^TCID_50_/mL) and the mixture was incubated at room temperature with rotation for 1 hour. Following the incubation the TCRV-NP mixture was added to Vero cells, seeded to 90% confluency, that were washed twice with PBS. The viral suspension was allowed to absorb for 1 hour at 37°C in 5% CO_2_. Following absorption, non-adherent virus was washed off using PBS, and DMEM supplemented with 2% FBS and 1% P/S was added to the cells which were then incubated at 37°C in 5% CO_2 _for 8 days, which is the time at which cytopathic effect was observed in nearly 100% of the cells infected with the untreated TCRV virus. Alternatively, samples from the 1 hour incubation of Ag-NPs with TCRV were also analyzed for size changes and agglomerate formation using DLS.

### Biocompatibility assay

Vero cells were seeded into 96-well tissue culture treated plates at a concentration of 50,000 cells/well. Twenty-four hours post seeding, the Vero cells were exposed to 10 or 25 nm Ag-NPs (either uncoated or PS-coated). The NPs were diluted to the various doses in DMEM+PS and sonicated with a probe sonicator for 30 seconds prior to exposure. At time points of 24 hours, 48 hours, and 8 days post exposure a standard MTS assay (Promega, Madison, WI) was performed to determine cell viability.

### Viral Inhibition Assay

Vero cells were exposed to Ag-NP-treated TCRV as described above. Following the 8-day incubation, progeny virus was harvested from the supernatants of infected cells and the cellular debris was removed from the viral suspension via centrifugation at 580 × g for 15 minutes. Virus titers were determined from the supernatants using a tissue culture infectious dose assay [21]. Briefly, Vero cells were seeded into 48-well tissue culture treated plates and grown to 90% confluency. The virus was serially diluted across six 10-fold dilutions and 100 μL aliquots per dilution (n = 8/dilution) were added to the cells. The virus was allowed to absorb for 1 hour at 37°C in 5% CO_2_. The cells were washed to remove non-adherent virions and DMEM containing 2% FBS and PS was added. The cells were incubated at 37°C in 5% CO_2 _for 8 days. Progeny virus titers are expressed as the reciprocal of the highest dilution that results in at least 50% of the wells displaying cytopathic effect [21]. Tissue culture infectious doses at 50% (TCID_50_) were compared between NP-treated and untreated TCRV infections.

### Confocal microscopy

TCRV was tagged with a carboxylic acid/succinimidyl ester mixed Alexafluor that excites at a wavelength of 488 nm by adding 10 μL of the AlexaFluor to 600 μL of concentrated virus in PBS and 400 μL of 0.2 M sodium bicarbonate and incubate at room temperature with rotation for 24 hours. The virus was recovered via centrifugation at 30,000 × g for 1 hour in 100 mM Glycine. TCRV~488 was incubated with Ag-NPs and then subsequently used to infect Vero cells for 4 hours, which was sufficient for intracellular detection but prior to diminishing of the fluorophore intensity. Hoechst was used for a nuclear stain and the amount of cell bound and internalized virus was examined using 15- 0.250 μm cell section collapsed z-stack images obtained via confocal microscopy (Becton Dickinson Pathway 435 spinning disc confocal microscope; BD, Franklin Lakes, NJ).

### Transmission Electron Microscopy

TCRV was incubated with the uncoated Ag-NPs at 50 μg/ml for 1 hour at room temperature with rotation. The resulting suspension was then used to infect Vero cells for 24 hours at 37°C in a 5% CO_2 _atmosphere. Several time-points were analyzed but the 24 hour seemed to have a lot of detectable intracellular virions. The cells were trypsinized, centrifuged at 500 × g, and fixed in a 2% paraformaldehyde/2.5% glutaraldehyde mixture for 2 hours. The cells were then washed and stained with 1% osmium tetroxide (EM Sciences) for 1 hour. The cells were washed again and stained with 2% phosphotungstic acid for 5 minutes. Following the staining the cells were dehydrated with the addition of successively higher concentrations of EtOH. After the sample had been dehydrated with 100% EtOH, a 1:1 mixture of LR white medium grade resin (EM Sciences) and 100% EtOH was added to the samples for 1 hour. The mixture was removed, and 100% resin was added. The resin was cured overnight in a vacuum oven at 60°C, -15 inHg. The embedded cells were thin sectioned on an ultramicrotome (Leica Ultracut) to ~50 nm and collected onto carbon-formvar-coated Cu TEM grids. Bright-field transmission electron microscope (TEM, Hitachi S) images were obtained for each sample at 100 kV.

### S segment real time PCR

Vero cells were exposed to Ag-NP treated TCRV as described above. Following 4-day incubation, the supernatant was removed from the infected cells and the cells were washed 2 times to remove non-internalized virus. The 4-day time-point was chosen since the TCRV RNA was easily detectable and produced in high amounts and there was no evidence of cytopathic effect yet, which could results in false-negative readings. RNA was prepared from the cells using the Qiagen RNeasy kit with the optional DNase treatment step. Concentrations of the RNA were determined using the Nanodrop ND-1000 spectrophotometer. Quantitative real time PCR (qRT-PCR) was performed by adding 100 ng of each RNA sample in triplicate to a one-step Sybr green real time PCR reaction mix (Invitrogen, Carlsbad, CA) and was analyzed using the Stratagene Mx3005p real time PCR machine (Stratagene, La Jolla, CA). The S gene segment was amplified using the following primers: F' tgtgttggctggcagat, R' aggagagtgaacaaagacat. β-actin was used for an internal control and was measured using published primers [22].

### Post-infection treatment with Ag-NPs

Vero cells were infected with untreated TCRV at 5 × 10^4 ^TCID_50_/mL for 1 hour at 37°C in 5% CO_2_. Following this incubation, the Vero cells were washed and DMEM+2% FBS was added to the cells and the cells were allowed to incubate for 8 days and progeny virus titer was determined as stated above. Either concurrently with addition of the virus (0 hour) or the DMEM/2% FBS (1 hour), or at various time-points thereafter, 10 nm uncoated Ag-NPs were added to the cells at 25 μg/ml. Changes in virus titer between the untreated Vero cells and the Ag-NP-treated cells were plotted.

### Statistical analysis

Data were expressed as the mean ± standard error of the mean (S.E.M.). The one-way ANOVA and the t-test (MS Excel; Microsoft Corporation, Redmond, WA or Prism 5 statistical and graphing software; GraphPad Software, Inc, La Jolla, CA) were used for the data analysis. The one-way ANOVA was used to determine the effect of test compound concentration on the mean number of treatments/well. The two-sample t-test was used to compare the average number of samples/well between the untreated positive controls and each treatment group. P ≤ 0.05 was used as the level for significance.

## Abbreviations

TCRV: Tacaribe virus; AG-NPS: silver nanoparticles; PS-AG: polysaccharide-coated silver nanoparticles; TCID_50_: tissue culture infectious dose at 50% infectivity.

## Competing interests

The authors declare that they have no competing interests.

## Authors' contributions

JLS wrote the manuscript, completed Figs 1, 2, 5 and 6 entirely and set up Figs 3, 4. RCM performed the TEM imaging and DLS experiment, and characterized the Ag-NPs upon receipt. LKB-S performed the confocal imaging. AMS wrote the TEM materials section. SMH is the laboratory principle investigator and helped design the methods. All authors have read and approved the manuscript.
